# Mechanistic Insights into Molecular Modifiers That Promote Urate Crystallization through Solute Assembly Regulation

**DOI:** 10.1021/jacsau.6c00140

**Published:** 2026-04-17

**Authors:** Qizan Chen, Si Li, Ryan Soucek, Jeffrey D. Rimer, Jeetain Mittal

**Affiliations:** † Artie McFerrin Department of Chemical Engineering, 14736 Texas A&M University, College Station, Texas 77843, United States; ‡ Department of Chemical and Biomolecular Engineering, 14743 University of Houston, Houston, Texas 77204, United States; § Department of Chemistry, Texas A&M University, College Station, Texas 77843, United States; ∥ Interdisciplinary Graduate Program in Genetics and Genomics, Texas A&M University, College Station, Texas 77843, United States

**Keywords:** ammonium urate crystallization, crystal growth modifier, riboflavin, molecular dynamics simulation, molecular self-assembly

## Abstract

Understanding crystallization modifier mechanisms remains a central challenge in crystal engineering, particularly for growth promoters whose modes of action are far less understood than inhibitors. Here, we investigate riboflavin (RF) as a crystallization promoter for ammonium urate (NH_4_HU) using combined microfluidic experiments and molecular dynamics simulations to elucidate its underlying mechanism. We discovered that RF reorganizes neighboring urate ions into growth-compatible coplanar conformations, contrasting with their naturally preferred growth-incompatible stacked arrangements. This identifies a solution-phase preassembly mechanism for enhancing crystal growth, distinct from classical monomer addition or traditional surface-based pathways. We found that the modifier’s ability to reorganize surrounding urate correlates with its aromatic ring size, explaining why the large RF framework exhibits unique promotion effects among known modifiers. Guided by detailed contact analysis between RF and urate ions, we rationally designed RF derivatives with enhanced promotion capabilities and experimentally validated their predicted performance, with the natural metabolite lumichrome showing a 40% growth enhancement compared to the 20% enhancement observed for RF. Our findings establish solution-phase preorganization as a viable mechanism for crystallization control.

## Graphical abstract

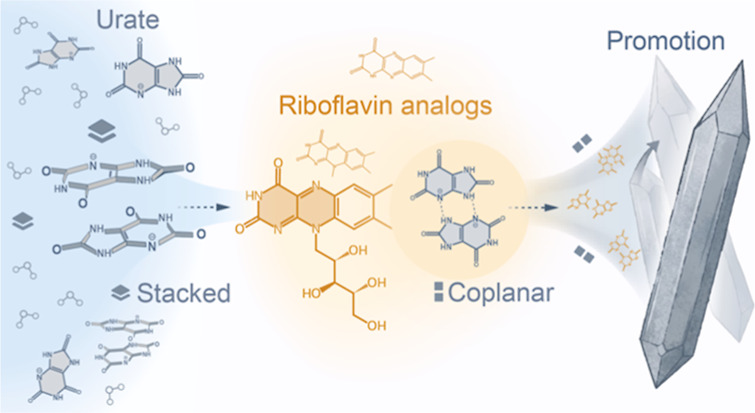

## 1 Introduction

Molecular crystallization is a precisely regulated process, spanning from the exquisite architecture of nacre and bone mineralization to the pathological precipitation of kidney stones and uric acid deposits in gout.^1–4^ This universal phenomenon extends to industrial domains where crystal structure (polymorph) and habit—the external morphology reflecting internal molecular organization—critically determines product performance.^5–7^ For instance, in the pharmaceutical industry, polymorphic control can determine the difference between therapeutic efficacy and toxicity,^8–10^ while in the development of advanced materials surface properties dictate functionality.^11,12^

At the molecular level, crystallization often emerges from the delicate interplay of weak intermolecular forces—hydrogen bonds, π–π stacking interactions, and halogen bonds. These competing weak forces, along with the influence of packing entropy, make crystal habit highly^13–15^ sensitive to the surrounding environment, including changes in acidity, temperature, or solvents.^16–18^ Chemical modifiers provide an elegant means to navigate this sensitivity, enabling fine-tuned control without drastic environmental changes, which is particularly important in biological contexts; they also span a wide spectrum of chemistries, from simple ions to complex macromolecules, thereby offering a broad design space for tailoring crystallization.^19–22^

The mechanistic landscape of crystal modification has been illuminated primarily through the lens of growth inhibition, where the classic Kossel terrace-step-kink (TSK) model provides a robust framework for understanding modifier action.^23,24^ In situ atomic force microscopy (AFM) combined with kinetic measurements has provided direct visualization of surface-based inhibitory pathways, transforming theoretical constructs into observable phenomena.^25–27^ These mechanisms include kink blocking, where modifiers occupy high-energy kink sites to impede step propagation; step pinning, which occurs when modifier molecules adsorb onto growth steps within critical distances to pin the advancing front.^19,21^ For crystals that grow by surface diffusion, modifiers that generate multisteps or step bunches reduce the rate of layer advancement.^28–30^ Studies have revealed these pathways can act synergistically or antagonistically.^31,32^ Additionally, inhibitors can operate by sequestering solutes to reduce supersaturation or by introducing lattice strain that destabilizes the crystal structure.^33–35^

In stark contrast, the molecular mechanisms underlying growth promotion remain far more elusive. This knowledge gap is due to the nature of promotion itself: it is not only observed less frequently than inhibition, but its mechanisms—often subtle, atomic-scale interactions—are highly system-specific, posing significant challenges to experimental validation and the development of generalizable models.^36,37^ These proposed mechanisms include:^38–41^ (i) creating local gradients in supersaturation by attracting solute to the crystal surface, (ii) reducing the surface free energy upon adsorption, and (iii) disrupting the local solvent structure to facilitate solute attachment. Relatively few modifiers are strictly growth promoters.^36–38^ The majority of reported cases show that promotion occurs at low modifier concentration and switches to growth inhibition at higher concentrations.^37,42,43^

All mechanisms discussed above underscore the critical role of molecular recognition at the crystal interface. This may include modifiers with functional groups positioned in specific patterns that bind preferentially to particular crystal surfaces.^44,45^ This principle is also evident in a distinct class of modifiers - imposters - that share certain structural elements with the crystallizing species, enabling them to be readily recognized and incorporated into the growing crystal lattice, yet their distinct structural features disrupt normal crystallization.^46^ For example, various urate imposters have been shown to inhibit ammonium urate (NH_4_HU) crystal growth,^36^ which has been extensively studied in part due to its occurrence in kidney stone formation in both humans and dolphins.^47–50^ Importantly, imposters can also arise endogenously in urate systems from molecules undergoing tautomeric transformations.^34^ Notably, while tautomerism is common in pharmaceuticals and bioactive compound libraries, its implications for crystallization remain largely overlooked.^51–53^

In the present study, we investigate riboflavin (RF) as an imposter of urate (HU^–^) affecting the crystallization of NH_4_HU (Figure 1A). While RF is a strong inhibitor of NH_4_HU growth at high concentrations, we found that it paradoxically functions as a promoter at low concentrations (Figure 1B). As previously mentioned, this concentration-dependent switch has been observed in other systems, but is especially intriguing for imposters, as it cannot be readily explained by conventional surface-based mechanisms of molecular recognition,^20^ suggesting a more complex mode of action.

**Figure 1:**
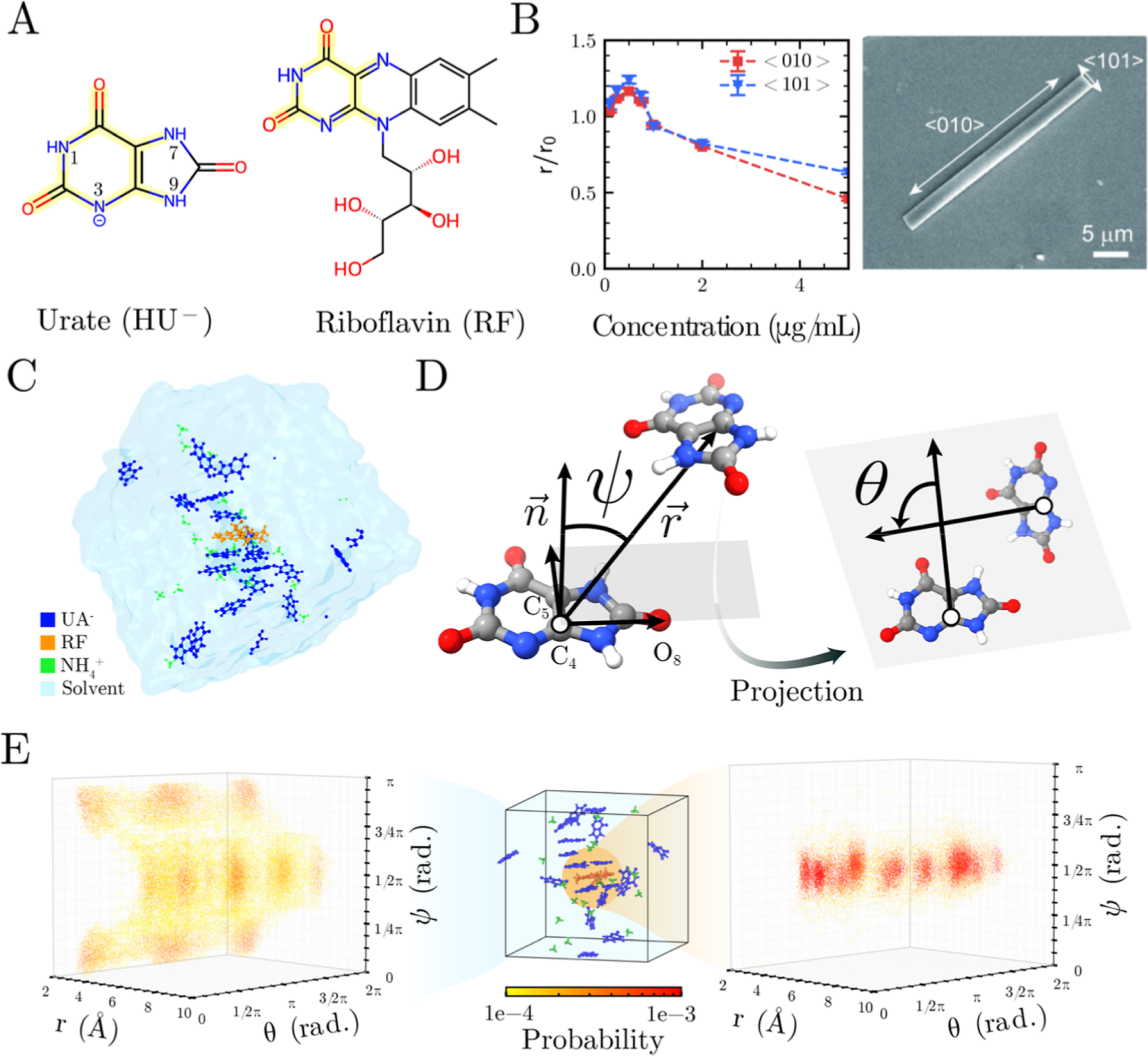
(A) Molecular structures of HU^–^ and RF with structurally analogous regions highlighted in yellow. (B) Left: scaled growth rates along ⟨010⟩ (red) and ⟨101⟩ (blue) in the presence of RF. The growth rates are scaled by *r*_0_, which is the value in the absence of modifier. Scaled growth rates >1 indicate growth promotion and <1 indicate growth inhibition. Data are the means over 10–15 crystals, error bars span two standard errors (SE), and dashed lines are guides to the eye. Right: scanning electron microscope (SEM) image of an NH_4_HU crystal with indexed directions. (C) Snapshot from the simulation of a single-RF (orange) system showing HU^–^ (blue) and NH_4_^+^ (green) in aqueous solution (water molecules are omitted for clarity). (D) Schematic diagram defining the intermolecular parameters *r*, θ, and ψ used to characterize the assembly in conformational space. (E) Representative 3D scatter plots of these parameters, colored by the probability of conformers appearing within different parameter intervals (details are provided in the “Methods” Section). The left panel shows HU^–^ dimers in the bulk solution, and the right panel shows those in the vicinity of the modifier (*R*_cut_ = 4.0 Å), highlighting the pronounced shift from stacked to coplanar conformations near RF, with the population shifting from low-probability (yellow) to high-probability (red) states around π/2.

One known inhibitory pathway of NH_4_UH growth involves the formation of RF–HU^–^ complexes.^33^ To explore whether such complexation might also be linked to promotion, we combined microfluidic experiments with molecular dynamics simulations. Simulations revealed that RF reorganizes neighboring HU^–^ ions into conformations more compatible with crystal patterns, leading us to propose a preorganization promotion mechanism. To test this hypothesis, we deductively designed novel RF derivatives based on simulation results and experimentally confirmed their predicted, enhanced promotion of NH_4_HU crystallization. Collectively, these findings support a solution-phase promotion mechanism for promoters, distinct from classical surface-acting pathways, and highlight the potential of such mechanisms to broaden applications of molecular imposters in crystal engineering. In doing so, our work deepens the mechanistic understanding of imposter modifiers and suggests that such mechanisms may extend key insights to ostensibly modifier-free systems.

## 2 Results and Discussion

### 2.1 Growth Promotion of NH_4_HU Crystals with RF

Microfluidic growth measurements revealed RF exhibits a concentration-dependent dual functionality in NH_4_HU crystallization. While it acts as an inhibitor at higher concentrations, RF clearly promotes growth at concentrations below 0.5 μg/mL (Figure 1B, left). This promotion was observed along both the ⟨010⟩ and ⟨101⟩ crystallographic directions, with growth rates increasing by approximately 20% relative to control (modifier-free) conditions. This promotion effect is more pronounced along the ⟨101⟩ direction, with the crystal growth directions shown in the scanning electron micrograph (Figure 1B, right).

Promotion at low concentrations implies that the mechanism does not require high surface coverage. Given that RF is known to form complexes with HU^–^ in solution, we hypothesized that RF might influence crystallization through solution-phase processes. This was motivated by our previous study where we showed that RF complexes multiple HU^–^ molecules in solution.^33^ To explore the hypothesis of a solution-phase process involving these complexes, we designed molecular dynamics (MD) simulations with a single RF molecule in the presence of HU^–^ and NH_4^+^_ ions in explicit aqueous solution (Figure 1C); detailed simulation protocols are provided in the Supporting Information Methods Section. This setup allows us to isolate the solution-phase effects of RF on HU^–^ assembly without complications from modifier–modifier interactions or competitive binding.

To establish a mechanistic connection between solution-phase self-assembly and crystal growth, we developed a quantitative framework based on the HU^–^ packing motifs present in the NH_4_HU crystal. Analysis of the NH_4_HU crystal structure reveals that HU^–^ molecules adopt two distinct interaction modes: π–π stacking arrangements with intermolecular distances of ∼4 Å, and coplanar hydrogen-bonded conformations where molecules lie in the same plane connected by directional hydrogen bonds with distances less than 2.5 Å (Figure S1). These two interaction modes correspond to different crystallographic directions: stacking occurs along ⟨010⟩ while coplanar hydrogen bonding is dominant within the (010) plane. The precise geometric requirements of these interactions create specific “fingerprints” that can serve as benchmarks for evaluating the compatibility of solution-phase assemblies with crystal incorporation (i.e., registry between the soluble complex and crystal lattice). We then designed three geometric parameters to quantify HU^–^ pair conformations in solution: the intermolecular distance *r*, the planarity angle ψ that distinguishes between stacked (ψ ≈ 0, π) and coplanar (ψ ≈ π/2) arrangements, and the relative orientation angle θ between intramolecular vectors (Figure 1D).

The MD simulations revealed a dramatic, RF-induced transformation in the local organization of HU^–^ ions. In the bulk solution, far from the modifier, HU^–^ dimers predominantly adopt stacked conformations as expected from their aromatic character, with ψ distributions centered around 0 and π (Figure 1E, left panel). However, HU^–^ molecules within 4 Å of RF show dramatically different behavior, with a strong preference for coplanar arrangements (ψ ≈ π/2) (Figure 1E, right panel). This is consistent with our previous findings: in solution, stacked urate conformations are energetically favored; and multiple urate molecules can form a complex with RF, preferentially localized near the aromatic ring of RF.^33^ Moreover, a recent study showed that guanine, which shares structural similarity with HU^–^, also tends to first stack into aggregates in solution, and then form ordered structures through a combination of stacking and hydrogen bonding.^54^ This reorganization represents a fundamental shift in solute–solute interactions, facilitated by RF’s ability to template multiple HU^–^ molecules simultaneously. Such modulation may resemble templating or seeding effects, potentially influencing the crystallization pathway.^55,56^

The significance of this stacked-to-coplanar reorganization is quantified in the 2D differential probability maps comparing bulk and RF-proximate HU^–^ pairs (Figure 2). The *r*-ψ map reveals a depletion of stacked conformations and a strong enhancement of coplanar arrangements near RF (Figure 2A). The *r*-θ map (Figure 2B) further shows that while the stacked conformations around 4 Å exhibit broad θ distributions, the coplanar conformations give rise to multiple narrow regions. This occurs because coplanar urate dimers can be further stabilized through hydrogen bonding. Moreover, urate molecules can form hydrogen bonds in multiple directional patterns, leading to several distinct probability peaks. Crucially, within this map, specific regions of enhanced probability align precisely with the geometric fingerprints of HU^–^ pairs found in the crystal structure.

**Figure 2:**
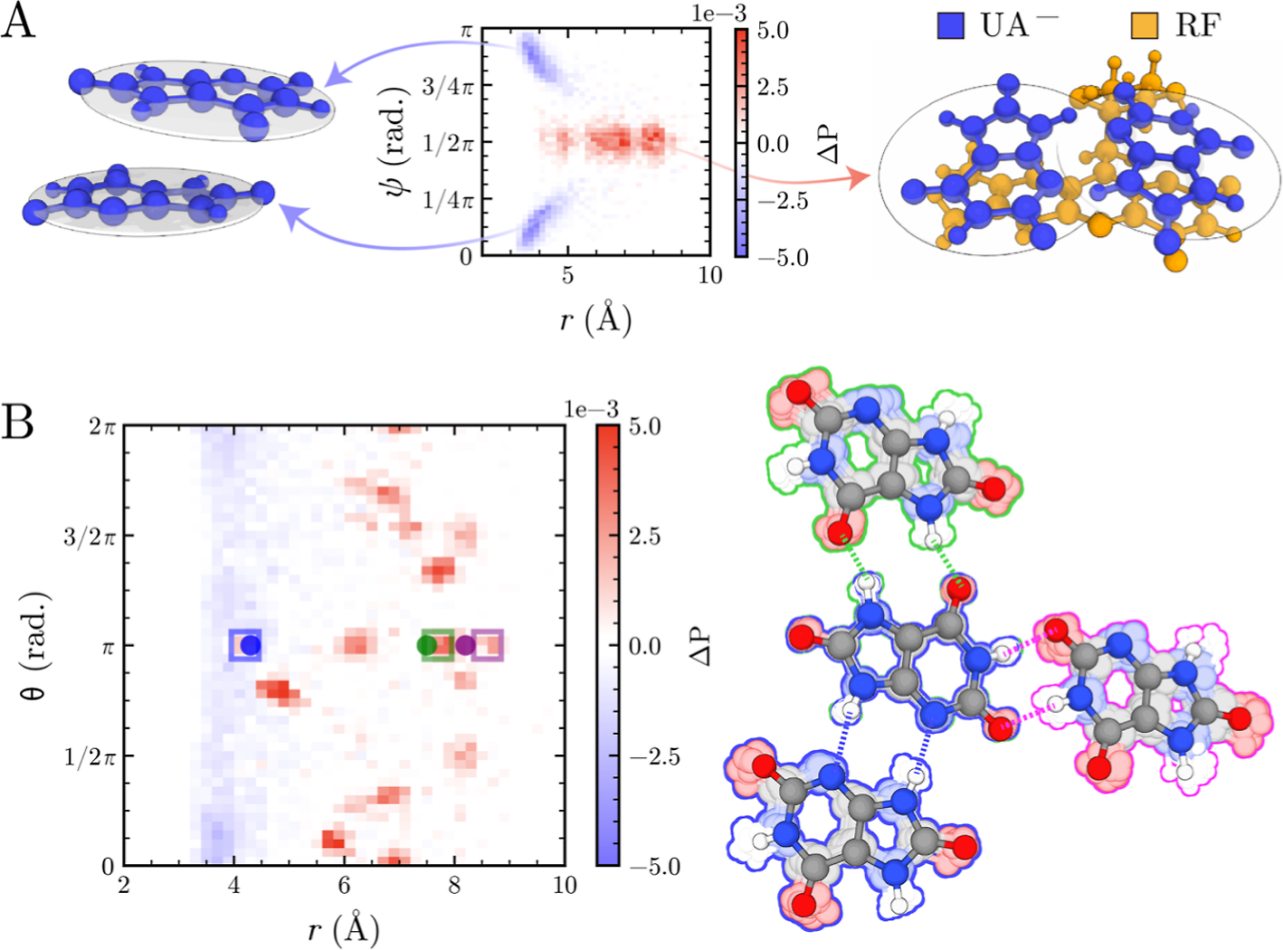
(A) Differences in the *r*-ψ probability distributions of HU^–^ in the bulk solution and near the modifier. The differences were obtained by subtracting the bulk solution distribution from that near the RF molecule. The color scale from blue to red indicates enhancement. The schematic on the left represents stacked HU^–^ dimers in the bulk, while the right represents coplanar HU^–^ dimers near RF. (B) Corresponding *r*-θ differential probability map with boxed regions indicating enhanced HU^–^ dimer conformations that match crystal-derived geometric fingerprints (dots), with the parameters of these regions listed in Table S1. On the right are HU^–^ dimer conformations from the crystal (opaque) together with an ensemble of HU^–^ dimers complexed with RF (RF hidden for clarity) obtained from simulations (semitransparent, 25 stacked), with dashed lines marking the corresponding hydrogen bonds. The colors for hydrogen bonds, conformations, and conformation ensembles are kept consistent.

Our previous work showed that HU^–^ molecules could stack in solution, which led us to further consider whether HU^–^ dimers directly participate in crystal growth; however, comparison of the experimental Raman spectra with DFT-calculated spectra (Figure S2) revealed that HU^–^ species in solution exist predominantly as monomers, and the measured step velocity of layer advancement (Figure S3) does not exhibit characteristics consistent with kinetic models of dimer incorporation reported by Vekilov and coworkers.^57^ Moreover, analysis of the stacked dimer geometries in MD simulations indicates that urate molecules preferentially adopt antiparallel stacking (Figure S4)—typical of heterocyclic aromatic rings.^58^ In contrast, HU^–^ molecules within the NH_4_HU crystal adopt parallel stacking (Figure S4), suggesting that the natural stacking tendency of HU^–^ in solution is crystallographically incongruent and therefore unlikely to directly contribute to crystal growth.

Consequently, we shifted our focus to the coplanar arrangements, proposing that RF’s ability to modulate surrounding HU^–^ ions into crystal growth-compatible coplanar conformations provides a promising mechanism for its promotion of crystal growth. This concept of solution-phase preorganization aligns with previous reports, such as for olanzapine, where molecules form crystal-compatible dimers that function as growth units.^57^ Compared to such spontaneous self-assembly, a modifier that actively enhances the crystal compatibility of dimers offers a more designable and tunable strategy for controlling crystallization. Moreover, this reveals the ability of RF to shift growth away from a classical mechanism involving monomer addition to a nonclassical pathway of dimer incorporation,^59^ which is not the native mode of crystal growth.

### 2.2 Molecular Basis of RF’s Selective Solution-Phase Templating Effect

Among all the HU^–^ imposters we have studied, RF is the only one that promotes growth at low concentration through solution-phase reorganization of HU^–^ assemblies.^33,36^ To understand what features make RF unique by comparing the solution assembly of HU^–^ around other modifiers, we conducted comparative simulations with four previously reported inhibitors: theophylline (TEP), methyluric acid (MUA), hypoxanthine (HPA), and purine (PUR). The ψ distribution analysis confirms that RF is uniquely effective at inducing coplanar HU^–^ arrangements, evidenced by the sharpest and most intense probability peak at ψ ≈ π/2 among all tested modifiers (Figures 3A and S5). Other imposters show varying degrees of influence, but none approach RF’s effectiveness in promoting coplanar conformations, consistent with experimental studies showing these molecules function solely as inhibitors over a broad range of concentrations.^36^ The specificity to promote coplanar conformations correlates strongly with aromatic ring size and surface area available for multimolecular templating, as revealed by spatial distribution function (SDF) analysis (Figures 3B and S6). RF’s expansive SDF isosurface readily accommodates two HU^–^ molecules side-by-side, creating the necessary scaffold for effective templating. In contrast, smaller modifiers like purine show restricted interaction volumes that limit their ability to organize multiple HU^–^ molecules simultaneously, while intermediate-sized molecules like theophylline show partial templating effects that are insufficient to significantly alter HU^–^ organization patterns (Figures S6A and S6B). Furthermore, at a lower SDF occupancy threshold, riboflavin still shows a stacked second-layer HU^–^ population, but this region is substantially smaller (Figure S6C), suggesting that templating from the first layer to the second layer is much weaker, similar to that observed for smaller modifiers.

**Figure 3:**
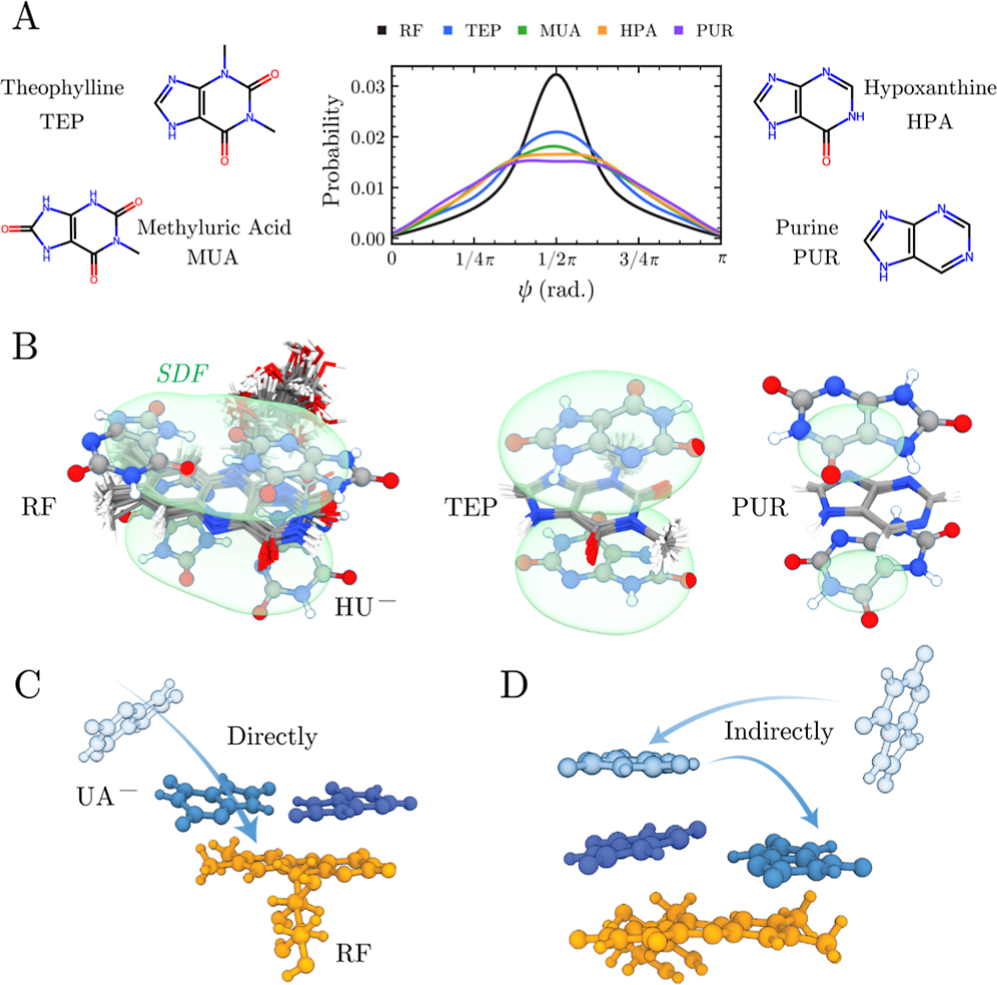
(A) Chemical structures of four previously reported NH_4_HU crystal growth inhibitors (imposters): theophylline (TEP), methyluric acid (MUA), hypoxanthine (HPA), and purine (PUR), with the middle panel comparing the ψ distributions of HU^–^ dimers in the vicinity of each modifier compared with that of RF (molecules are color coded by functionality). (B) SDF isosurfaces (transparent green) of HU^–^ distributions around each aligned modifier at an occupancy value of 0.4 (see the Methods Section in the Supporting Information). Modifier molecules are shown in stick representation (25 stacked conformations), and HU^–^ molecules in ball-and-stick representation are added for reference to the width of the SDF isosurfaces. (C,D) Simulation snapshots illustrating two pathways by which RF captures two HU^–^ molecules: (C) direct adsorption of two separate HU^–^ molecules (see Movie S1) and (D) indirect capture via initial stacking of a free HU^–^ with an adsorbed HU^–^ followed by transfer to the RF aromatic ring (see Movie S2).

Dynamic trajectory analysis further revealed two distinct pathways by which RF captures and organizes HU^–^ molecules (Figure 3C,D, and Movies S1 and S2). The first is a direct pathway, where two HU^–^ molecules separately adsorb onto the same side of the RF aromatic core. The second pathway begins with conventional π–π stacking of HU^–^ onto an already-adsorbed molecule, followed by reorganization into coplanar hydrogen-bonded conformations. This indirect pathway demonstrates RF’s ability to not only attract HU^–^ molecules but also actively restructure their mutual interactions once they are within the templating environment. Additionally, hydrogen bonding between HU^–^ molecules around RF remains highly dynamic, with rapid transitions between different hydrogen-bonding modes (Movie S3), which explains the appearance of multiple valleys in the *r*-θ distribution (Figure 2B).

A detailed contact analysis reveals the specific molecular interactions responsible for RF’s templating effect (Figures 4 and S7). The ribityl side chain of RF, particularly hydrogen H_13_ (Figure 4, middle panel), shows high contact frequency with HU^–^ acceptor atoms (N_3_, O_2_, O_6_, O_8_), indicating strong attractive interactions that contribute to the overall binding affinity; however, compared to HU^–^ molecules not interacting closely with H_13_ (Figure 4, lower panel), those near H_13_ (Figure 4, upper panel) show significantly fewer crystal-conforming arrangements, suggesting that H_13_ hydrogen bonding with HU^–^ affects the way HU^–^ molecules form crystal-compatible hydrogen bonds with each other. This analysis suggests that the side chain, while contributing to overall binding affinity through strong hydrogen-bonding interactions, may actually hinder optimal preassembly by stabilizing conformations that are not conducive to crystal growth.

**Figure 4:**
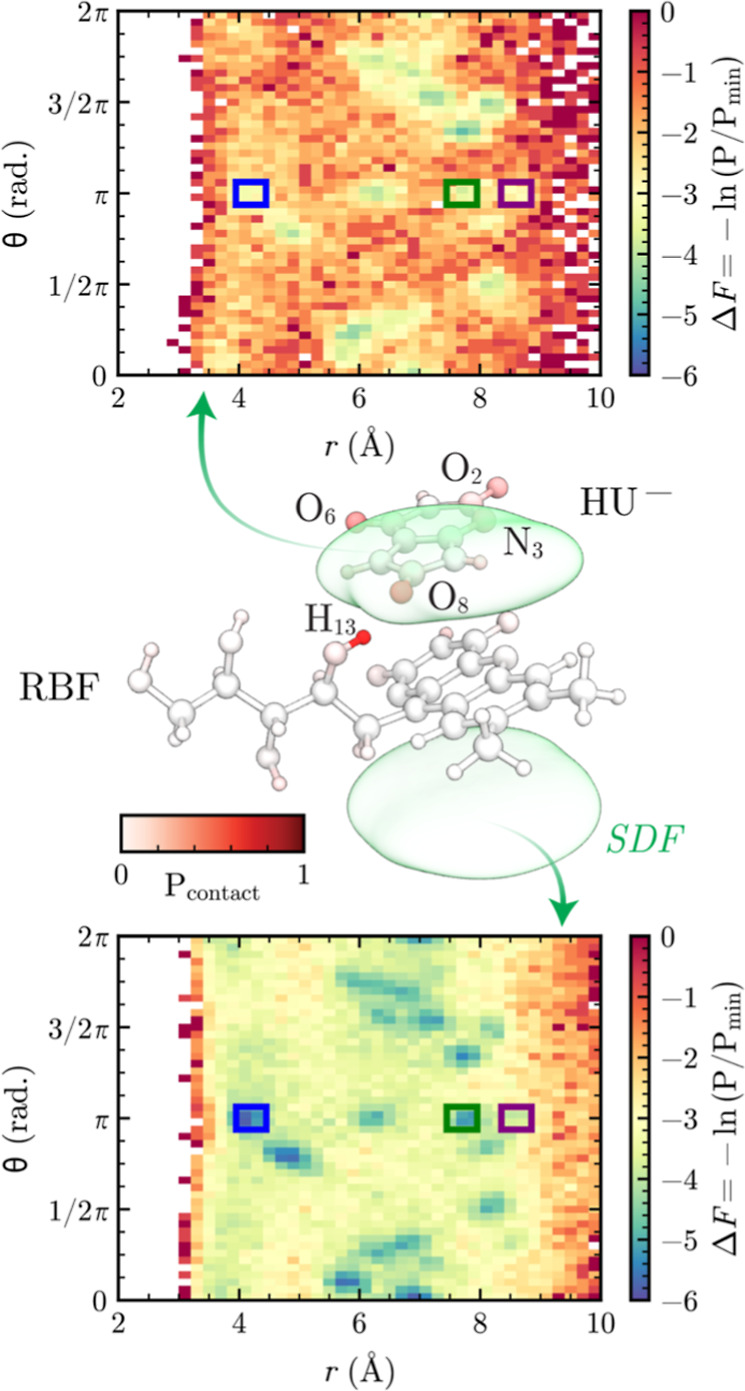
Hydrogen-heavy atom contact frequency between RF and HU^–^ (middle) and orientational free-energy analysis of HU- dimers near RF (upper and lower). Middle panel, hydrogen-heavy atom contact frequency (distinguished by color intensity) between RF and HU^–^ (*R*_cut_ = 2.5 Å), with major contact atoms labeled and the SDF isosurfaces as in Figure 3. Upper and lower panels: the *r*-θ free-energy maps of HU^–^ molecules that contact (upper) the side-chain hydrogen H_13_ of RF and (lower) nonside-chain hydrogens of RF and their neighboring HU^–^ molecules.

### 2.3 Rational Design of Potentially Superior Promoters

We systematically designed and simulated three RF derivatives as potential modifiers of NH_4_HU crystals through structural alterations: lumiflavin (LUF), which lacks the terminal hydroxyl groups of the ribityl chain; lumichrome (LUC), which lacks the entire ribityl chain; and alloxazine (ALO), which additionally lacks two methyl groups from the aromatic core (Figure 5A). These molecules all exhibit biocompatibility and have been explored as potential therapeutics for other diseases.^60–63^ The series allows us to test specific hypotheses about the roles of different molecular components in the templating mechanism. The probability analysis of crystal-conforming HU^–^ dimers reveals that both lumichrome and lumiflavin exceed RF’s performance in promoting crystal growth-compatible conformations, validating our hypothesis that optimizing the side chain can enhance the promotion of growth-compatible dimers. Alloxazine shows reduced activity, again emphasizing the importance of having a sufficiently large aromatic core for effective templating (Figures 5B, S8 and S9). We also found that even modifiers without significant promotion effects locally influence HU^–^ aggregation. This suggests that growth promotion requires a threshold degree of solute reorganization, beyond which crystal growth-compatible assemblies can effectively couple to surface incorporation at growth sites.

**Figure 5:**
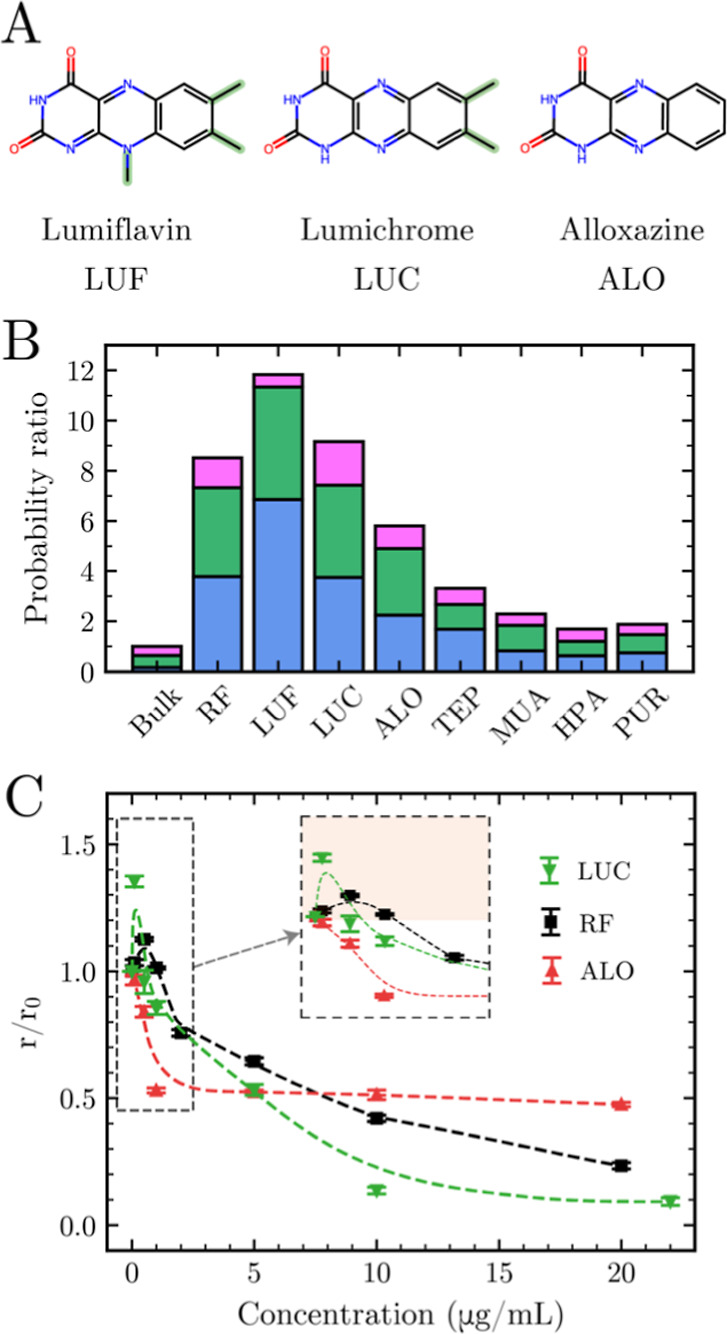
(A) Chemical structures of three designed modifiers: lumiflavin, lumichrome, and alloxazine, with structural differences highlighted in green. (B) Probability of crystal-conforming urate dimers for HU^–^ near each modifier and in the bulk, where crystal-conforming regions are defined directly from the NH_4_HU lattice (see Table S1), normalized to bulk proportions. The colors and crystal-conforming regions are consistent with Figure 2, and their parameters are detailed in Table S1. (C) Experimental scaled growth rates of NH_4_HU crystals along the ⟨010⟩ direction in the presence of different concentrations of the modifiers: lumichrome (green), riboflavin (black), and alloxazine (red). Growth rates *r* and *r*_o_ measured in the presence and absence of modifiers, respectively, were obtained from microfluidics using previously reported protocols.^33^ Data show means over 10–15 crystals, error bars represent standard errors (SE), and dashed curves are eye guides. The dashed box highlights the growth promotion regime, with the inset providing an enlarged view for RF and LUC.

Considering drug pricing and accessibility for potential therapeutic applications, we experimentally tested lumichrome and alloxazine as representative derivatives. Lumichrome exhibits enhanced promotion (40% growth rate increase) compared to RF (20% increase), while alloxazine shows almost no promotion activity (Figure 5C). The excellent agreement between computational predictions and experimental results validates the proposed solution-phase preorganization mechanism and also motivates future studies on LUF, which is a more expensive RF variant. Importantly, the concentration-dependent profiles show that modifiers with stronger promotion at low concentrations also exhibit more potent inhibition at higher concentrations. This correlation suggests that the modifiers’ ability to promote crystal-conforming HU^–^ patterns may be associated with stronger binding between the modifier and the crystal surface, thereby enhancing molecular recognition and helping explain the observed promotion at low concentrations and inhibition at higher concentrations. This again reflects the effectiveness of molecular recognition as a fundamental theoretical framework for predicting a modifier’s capability to promote crystal growth.

## 3 Conclusion

In summary, we present a combined computational and experiment study showing how crystal growth modifiers can preassemble growth-compatible dimers in solution as a means of promoting crystal growth. This solution-phase preassembly mechanism represents a fundamental departure from traditional crystal growth modifier models that focus exclusively on modifier-surface interactions. Because this mechanism operates entirely in solution, it provides a generalizable route for controlling crystallization by reshaping solute assembly prior to incorporation, rather than by direct surface binding. In this context, RF functions as a molecular template that directs solute molecules to aggregate into crystal growth-compatible conformations, thereby facilitating faster solute incorporation. This mechanism elegantly explains the experimentally observed concentration dependence where, at low concentrations, RF primarily acts to preassemble solute molecules in conformations that are in registry with the crystal lattice to enhance growth; and at higher concentrations, excessive RF molecules preferentially interact with crystal surfaces, thereby inhibiting growth through both thermodynamic effects (by reducing supersaturation) and kinetic effects arising from directed RF–crystal interactions.^33^ A switch from monomer to dimer addition also reveals the ability of RF to regulate the mechanism of crystallization from classical to nonclassical, respectively.

The successful rational design and experimental validation of an enhanced promoter underscores the power of our integrated experimental-computational approach. In an era where data-driven and high-throughput screening methods are increasingly prevalent in molecular design, the *de novo* design of crystallization modifiers remains a formidable challenge, often yielding limited mechanistic insight. Our work serves as a testament to the enduring value of a classical mechanism-driven strategy. By using experimental observations to guide targeted simulations, we have demonstrated that a deep understanding of the underlying physical processes can yield reliable and translatable principles for molecular engineering, an outcome not always achievable through purely empirical, data-driven approaches.

The framework established here—identifying and engineering solution-phase preorganization—offers a new and potentially more accessible pathway for creating crystallization modifiers. Modulating interactions in the solution phase may prove more tractable than engineering the complexities of a solid–liquid interface. This approach opens new avenues for controlling crystal formation in pharmaceuticals, materials science, and biomineralization, transforming how we design molecules to direct the assembly of complex crystalline systems.

## Supplementary Material









## Notes

Experimental methods for crystal preparation, microfluidic assays, Raman spectroscopy, kinetic-model analysis, molecular dynamics simulations, and DFT calculations, with associated Figures and Tables (PDF)
Movie S1 (GIF)
Movie S2 (GIF)
Movie S3 (GIF)
