# Supplementary material for: Mechanistic Insights into Molecular Modifiers That Promote Urate Crystallization through Solute Assembly Regulation
Source: JACS Au. 2026 Apr 17;6(5):2819–26. doi: 10.1021/jacsau.6c00140 (PMC13213513; doi:10.1021/jacsau.6c00140)
Supplement: Supplementary file 1 [file au6c00140_si_001.pdf]

Supporting Information for

Mechanistic Insights into Molecular Modifiers that Promote Urate Crystallization Through Solute Assembly Regulation

Qizan Chen[1,†], Si Li[2,†], Ryan Soucek[1], Jeffrey D. Rimer[2], Jeetain Mittal[1,3,4*]*

[1] Artie McFerrin Department of Chemical Engineering, Texas A&M University, College Station, TX 77843, USA

[2] Department of Chemical and Biomolecular Engineering, University of Houston, Houston, TX 77204, USA

[3] Department of Chemistry, Texas A&M University, College Station, TX 77843, USA

[4] Interdisciplinary Graduate Program in Genetics and Genomics, Texas A&M University, College Station, TX 77843, USA

† These authors contributed equally

*Corresponding authors: jrimer@central.uh.edu, jeetain@tamu.edu

Methods

Materials. The following chemicals were purchased from Sigma Aldrich (St Louis, MO): uric acid anhydrous (≥99%), ammonium hydroxide (28% NH_3 in H_2O, ≥99.99%), riboflavin (RF, analytical standard), lumichrome, and alloxazine (96%). Poly-(dimethylsiloxane) (PDMS, 184 SIL ELASTOMER) was sourced from Dow. All reagents were used as received. Deionized (DI) water, used in all experiments, was purified with an Aqua Solutions RODI water purification system (18.2 MΩ).

Preparation of NH_4HU Crystals. NH_4HU crystals for *in situ* microfluidic were prepared using a two-step process. In the first step, seed crystals were formed via spontaneous crystallization by incubating a 100 mL solution of 7.5 mM ammonium urate (pH 11) at 21 ± 1 °C for 48 h. In the second step, the resulting seed crystal suspension was transferred to a 500 mL glass bottle containing 400 mL of fresh NH_4HU growth solution (5.5 mM ammonium urate, pH 11). This mixture was incubated for 7 days for the further growth of needle-like crystals. The crystals were harvested by filtration, resuspended in fresh 5.5 mM ammonium urate growth solution (pH 11), and incubated further. Repeating this procedure for 3–5 cycles resulted in crystals with 10−50 mm in length and 1-2 mm in width. NH_4HU solutions were prepared by dissolving precise amounts of anhydrous uric acid powder in DI water, followed by pH adjustment to 10.2 using 28 wt % $NH_3 \cdot H_2O$ under rapid agitation at 72 °C. The pH of solution was measured using an Orion Star A215 pH benchtop meter with a ROSS Ultra electrode (8102BNUWP).

Microfluidic Assays. A microfluidic platform, as detailed in our previous study, was employed for *in situ* analysis of crystal growth kinetics(1). The device, fabricated from poly-dimethylsiloxane (PDMS) bonded to a glass slide (7.5 cm×5 cm), featured with a cuboid chamber with dimensions of 1.2 cm×1.2 cm×0.8 cm. NH_4HU crystals, pre-deposited on a glass piece (1 cm×1 cm), were positioned at the chamber's center. This configuration facilitated real-time monitoring of crystal growth using an inverted microscope (Leica DMi8 instrument). Growth solutions containing 8 mM ammonium urate (pH 7.4±0.1) and varying concentrations of modifiers (0-50 mg/mL) were introduced to study the

impact of modifiers on NH_4HU crystal growth kinetics. A dual syringe pump (CHEMYX Fusion 4000) delivered the growth solutions continuously to the microfluidic device at a flow rate of 6 $mL \cdot h^{-1}$ for at least 120 mins. To mitigated light induced effects, syringes were wrapped by aluminum foil. Time-elapsed optical micrographs of crystals were captured and analyzed using Image J 64 to quantify incremental changes in crystal size along the <010> and <101> directions. Growth rates were determined by applying linear regression to the time-dependent crystal size data, and average growth rates were calculated from measurements of 15 to 20 individual crystals.

Raman spectroscopy. Raman spectra of urate solutions were collected using a HyperFluxPRO Plus Raman spectrometer (785 nm excitation) equipped with a Tornado crystalline probe (Hudson). The instrument employs a 785 nm laser operated at 200 mW, which was directed into the solution contained within a Technobis Crystalline reactor cell. Spectral acquisition was performed using the Tornado Spectral Software, with each spectrum averaged over 100 scans and an exposure time of 150 ms per scan. Aqueous solutions of sodium urate with concentrations ranging from 0.5 to 20 mM were prepared by dissolving the appropriate amount of sodium urate in 5 mL of deionized water. Each solution was filtered, transferred into an 8 mL quartz vial, and placed in the Crystalline reactor cell equipped with the Raman probe for in situ spectral analysis.

Kinetics models for step growth. Kinetic laws correlating the step velocity v with the total solute concentration C have been reported for four distinct cases, classified according to (i) the dominant solute species in solution (monomer or dimer) and (ii) the form of the growth unit (monomer or dimer) incorporated at the crystal steps. These four kinetic models provide a framework for linking solution speciation with step-growth mechanisms(2). Based on Raman spectral analysis (Figure S2), urate in aqueous solution exists predominantly in the monomeric form under the conditions studied. Accordingly, only the two kinetic models that assume monomer-dominated solution species were considered here:

(1) Monomer incorporation, for which the v-C relationship is given by

$$v = \frac{D_M \Omega_M}{\Lambda_M}(C - C_e) \tag{S1}$$

(2) Dimer incorporation, for which the v-C relationship is given by

$$v = \frac{D_D \Omega_D}{\Lambda_D}(C^c - C_e^2) \tag{S2}$$

Here D/Λ represents the kinetics coefficient and Ω is the volume of the incorporating species. The subscripts M and D denote monomer and dimer, respectively. The step velocities of NH_4HU $(20\bar{2})$ surface measured at pH 11 were fitted using Equation S1 and S2, and the corresponding fitting results are presented in Figure S3.

Molecular Dynamics (MD) Simulations. To investigate the self-assembly behavior of uric acid (HU) molecules and their interactions with modifiers, we performed all-atom molecular dynamics simulations using GROMACS version 2020.6(3). The molecular force field for HU^- and modifiers was based on the second-generation generalized AMBER force field (GAFF2), with parameters generated using the

ANTECHAMBER tool and atomic charges assigned using the AM1-BCC method(4). Topology files in GROMACS format were prepared using the ACPYPE package, and the TIP3P water model was employed for the simulations.

During the simulations, van der Waals interactions were truncated at a cutoff distance of 1 nm, and periodic boundary conditions were applied in all three dimensions. Coulomb interactions were calculated using the particle mesh Ewald method, also with a cutoff distance of 1 nm. To optimize simulation efficiency, hydrogen mass repartitioning was applied, increasing the mass of non-solvent hydrogen atoms to 3.024 while correspondingly reducing the mass of the associated heavy atoms(5, 6). Additionally, all bond lengths involving hydrogen atoms were constrained using the LINCS algorithm to eliminate fast degrees of freedom associated with bond vibrations, allowing for a larger time step. A leap-frog integrator was employed with a time step of 4 fs.

Initial structures were generated using the GMX GENBOX command, with water molecules randomly replaced by the studied small molecules. All systems were first optimized via energy minimization to ensure the maximum force did not exceed 1000 kJ/mol/nm. This was followed by 4 ns of NVT and NPT equilibration simulations to relax the molecular configurations. Finally, production simulations were performed under isothermal (300 K) and isobaric (1 bar) conditions for 200 ns to analyze the self-assembly behavior. For the modifier simulations, a 4 × 4 × 4 nm^3 simulation box containing a single modifier molecule surrounded by HU^- molecules at a density of 0.3 molecules/nm^3 was constructed. At this temperature and density, HU^- molecules were not expected to exhibit significant aggregation. Five independent simulations were carried out, with trajectory data saved every 0.1 ns. In the simulations, all molecules were unconstrained. The spatial distribution function (SDF) of atoms was computed after applying translational and rotational fittings based on the root-mean-square deviation (RMSD) of a centered modifier. The VOLMAP tool in VMD was then used to calculate the molecular occupancy on a spatial grid with a resolution of 0.1 Å(7). At each frame, grid points within the van der Waals radius of any particle were marked as occupied, and the overall occupancy at each point was obtained by averaging over all trajectory frames. The resulting spatial data were visualized in ChimeraX using an isosurface value of 0.4(8).

To assess whether the simulation length was sufficient, the contact lifetime between the modifier and surrounding HU^- molecules was quantified using a bond-lifetime autocorrelation analysis. A binary contact function, H(t), was defined for each modifier-HU^- pair, where H(t)=1 when the HU^- molecule was within 4 Å of the modifier and H(t)=0 otherwise. The time correlation function was calculated as $C(\tau) =< H(t_0\ )Ht_0 +\tau >$ using multiple time origins over five independent trajectories, and the characteristic contact lifetime was obtained from $\tau = \int_0^{\infty} C(\tau)d\tau$.The resulting average contact lifetime was 3.89 ± 0.20 ns, which is much shorter than the 200 ns production trajectory length and supports that the relevant local association and dissociation dynamics were well sampled. Further, an unconstrained 100 ns NPT simulation of the NH_4HU crystal was performed at 300 K and 1 bar to assess the ability of the force field to preserve the experimental crystal structure. The lattice parameters differed from the experimental values by approximately 5% for the a-direction and 2% for b- and c-directions, and the crystal packing remained intact throughout the simulation (Figure S11). Within this level of deviation, we consider the force field sufficiently accurate for the present

purpose (9–11).

The probability of each parameter was determined by performing histogram calculations and normalizing the counts by the total number of samples. For the 3D, 2D, and 1D analyses, 30, 50, and 100 bins were used within each parameter range, respectively. The continuous 1D probability profiles were obtained by applying a Gaussian kernel density estimation (KDE) to the discrete histogram data, using the bin width as the kernel bandwidth.

Density Functional Theory (DFT) Calculations. Raman spectra were obtained using density functional theory (DFT) calculations at the M06-2X level of theory with the 6-311+G(d,p) basis set, consistent with our previous work. Initial conformations were exported from molecular dynamics (MD) trajectories that represent characteristic parameter distributions. All calculations were performed using the Gaussian 09 software package(12), employing the SMD solvation model to account for aqueous-phase effects. Then, each conformation was geometry-optimized to ensure the absence of vibrational modes corresponding to imaginary frequencies. The Raman spectra continuous intensity profiles were generated using Multiwfn by applying Lorentzian broadening with a half-width of 8 cm^{-1}(13).

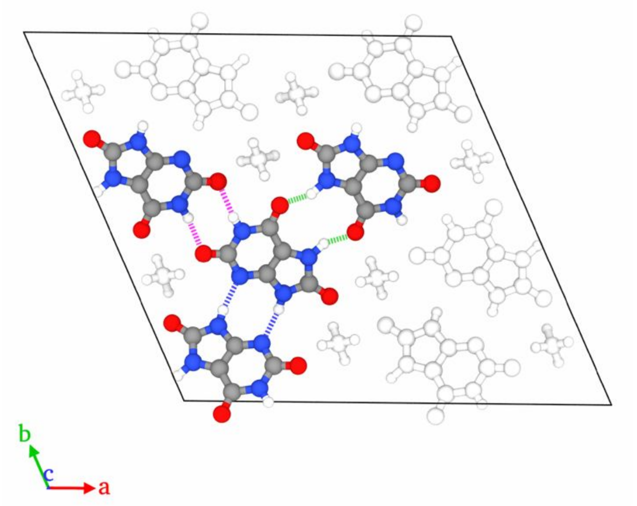

Figure S1. Crystal structure of NH_4HU viewed along the (010) plane, with the three hydrogen-bonding motifs highlighted in different colors. Atoms unrelated to these hydrogen bonds are shown in white for clarity. The crystallographic axes (a, b, c) are indicated at the bottom left.

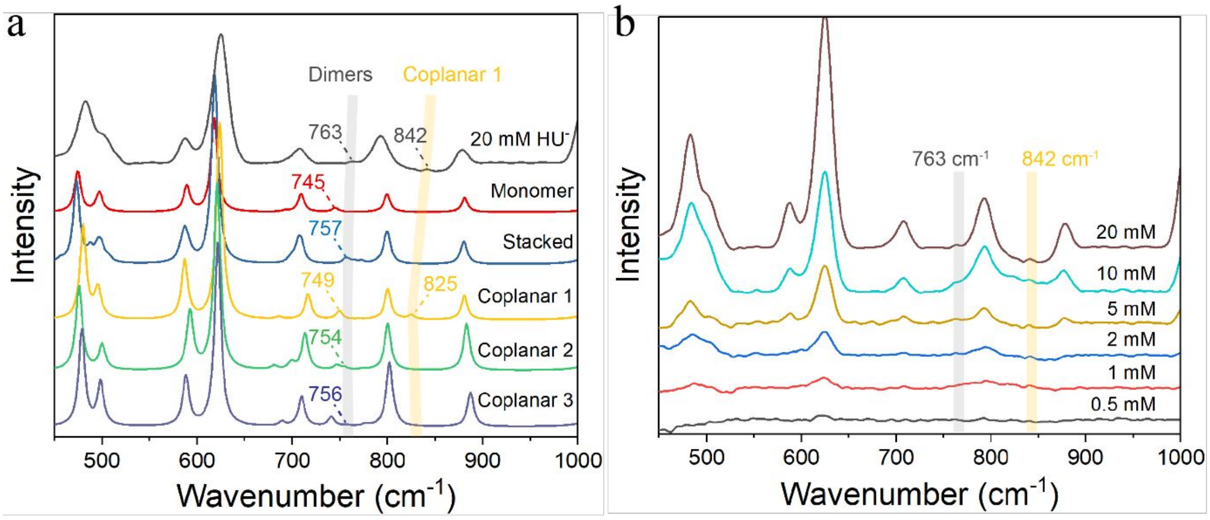

Figure S2. Computable and experimental Raman spectra of urate. (a) Raman spectrum of 20 mM HU^- in aqueous solution (black) and computable Raman spectra of HU^- monomer and dimers (stacked and coplanar). In the stacked conformation, the two HU^- molecules adopt opposite orientations (as in Figure S4, with an angle near π). The coplanar conformations 1, 2, and 3 correspond to those highlighted in pink, green, and blue, respectively, in Figure 2. The experimental peak at 763 cm^{-1}, corresponding to the characteristic peaks of HU- dimers (stacked dimer at 757 cm^{-1}, coplanar dimers 1-3 at 749cm^{-1}, 754cm^{-1}, and 756 cm^{-1}, respectively), and the peak at 842 cm^{-1} (matching coplanar dimer 3 peak at 825 cm^{-1}) together confirm the presence of these dimeric species in solution. (b) Experimental Raman spectra of HU^- aqueous solution at various concentrations. The increasing peak intensities at 763 and 842 cm^{-1} with concentration indicate the fraction of dimers rises with increasing HU^- concentration. Comparison between experimental and computed spectra indicates that HU^- dimers are present only in a small fraction in solution and are predominantly in stacked conformations that are inconsistent with the NH_4HU crystal.

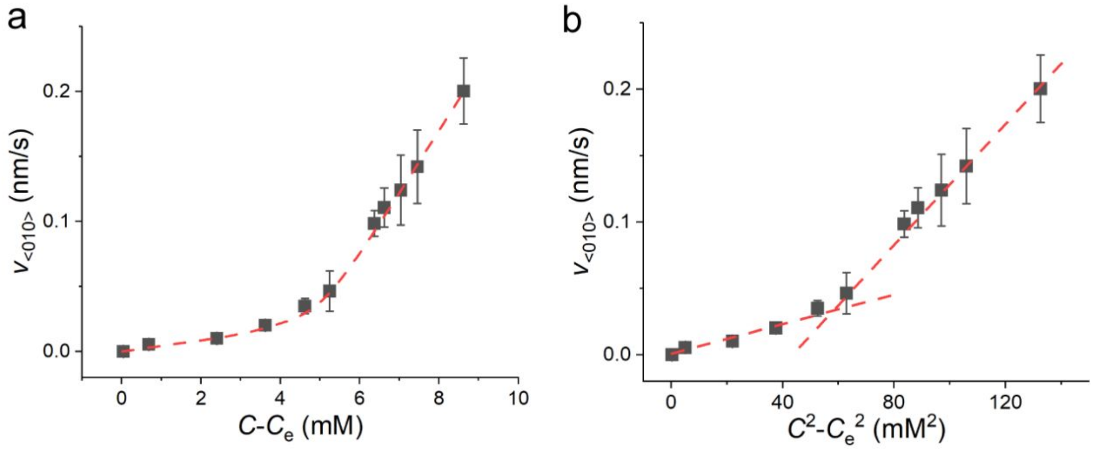

Figure S3. Step velocity (v) of layer advancement on the NH_4HU $(20\bar{2})$ surface at pH 11 as a function of (a) C-C_e and (b) C^2-C_e^2, where C is the solution concentrations and C_e is the solubility of NH_4HU crystals at pH 11 (C_e = 3.38 mM). Data are reproduced from Tang et al.(1, 14). Due to the presence of HU^- minor tautomer and the ionic isomer (U^{2-}) in solution at pH 11, interpretation of these curves is nontrivial; therefore, the observed superlinear correlation between v and (a) C-C_e and (b) C^2-C_e^2 does not exclude the possibility of growth via monomer or dimer incorporation. As shown in Figure S2, the concentration of dimers is markedly lower than monomers, suggesting the latter are likely the predominant growth unit for NH_4HU crystallization.

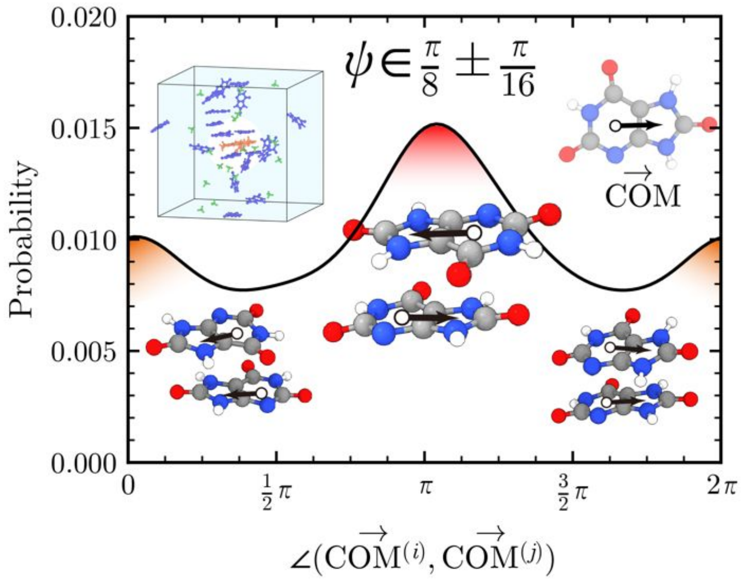

Figure S4. Distribution of the angle between the intramolecular center-of-mass vectors ($\overrightarrow{\mathrm{COM}}$) of the two rings in stacked HU^- dimers in the bulk solution (selected as those with $\psi \in \pi/8 \pm \pi/16$; ψ definition as in Fig. 1). The inset on the left highlights the bulk condition, while the right illustrates the $\overrightarrow{\mathrm{COM}}$ defined from the six-membered ring toward the five-membered ring. Representative snapshots of HU^- dimers with different $\overrightarrow{\mathrm{COM}}$ angles are shown along the plot.

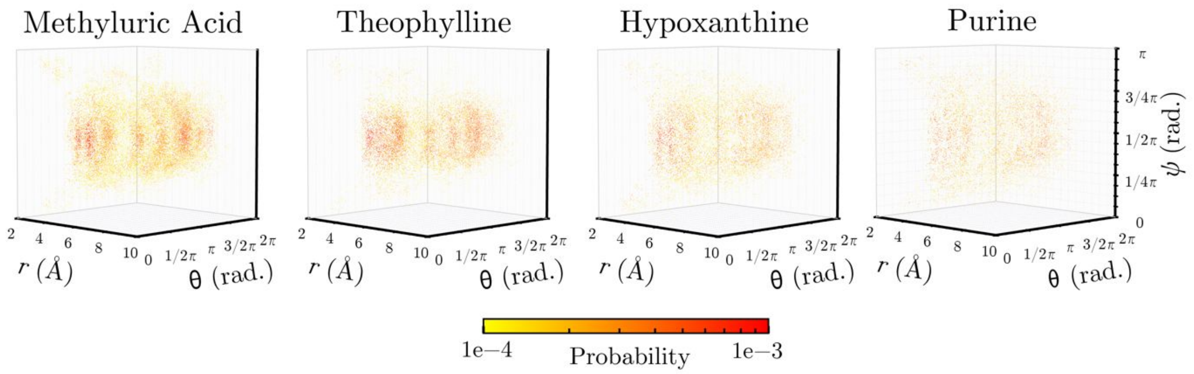

Figure S5. Representative 3D scatter plots of the parameters r, θ, and ψ, colored by probability, for HU^- dimers in the vicinity of methyluric acid, theophylline, hypoxanthine, and purine.

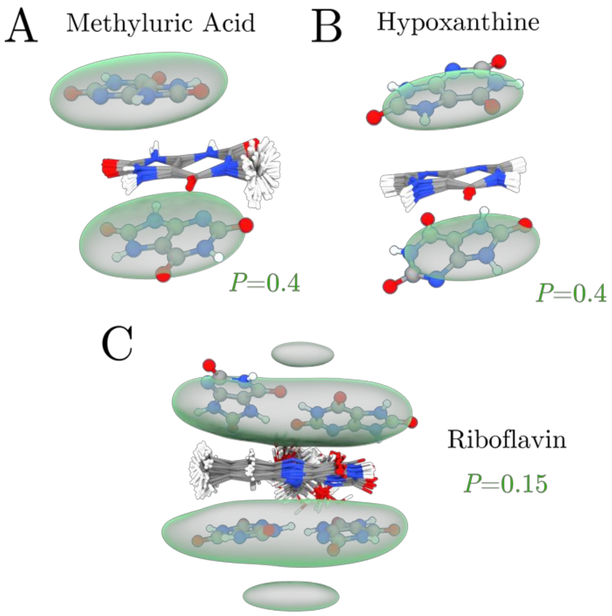

Figure S6. Spatial distribution function (SDF) isosurfaces (transparent green) of HU^- distributions around modifiers. (A,B) Methyluric acid and hypoxanthine at an occupancy value of 0.4. (C) Riboflavin at an occupancy value of 0.15. Modifier molecules are shown in licorice representation (25 stacked conformations), and HU^- molecules are shown in ball-and-stick representation.

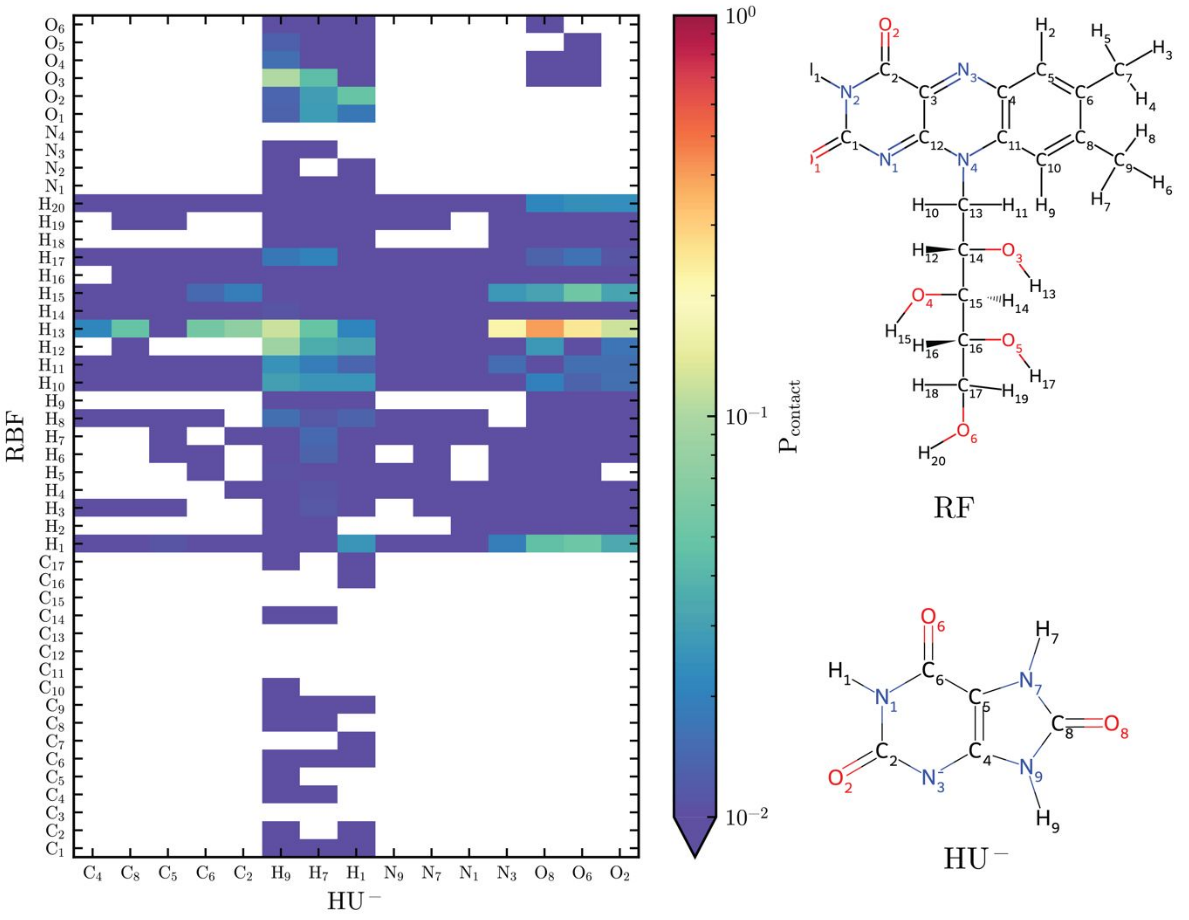

Figure S7. Atom–atom contact frequency map between RBF and HU$^-$, with atomic labels for RBF (top right) and HU$^-$ (bottom right) indicated in the corresponding molecular structures.

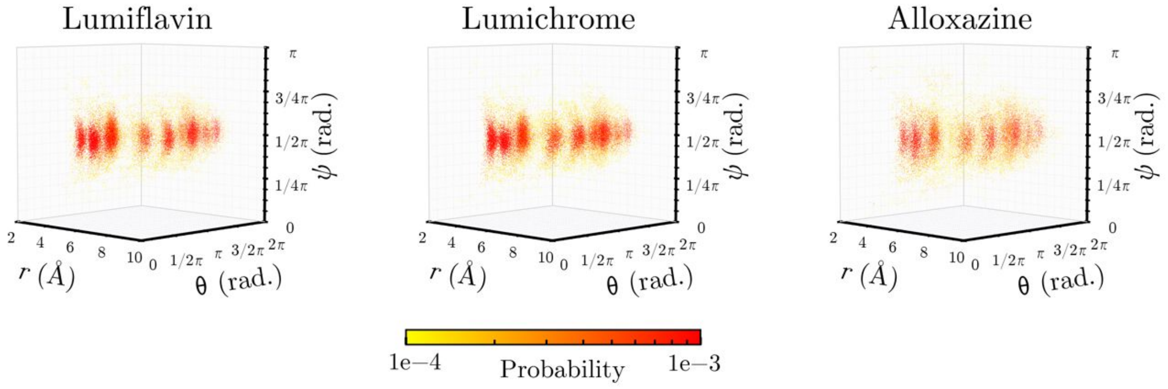

Figure S8. Representative 3D scatter plots of the parameters r, θ, and ψ, colored by probability, for HU$^-$ dimers in the vicinity of lumiflavin, lumichrome, and alloxazine.

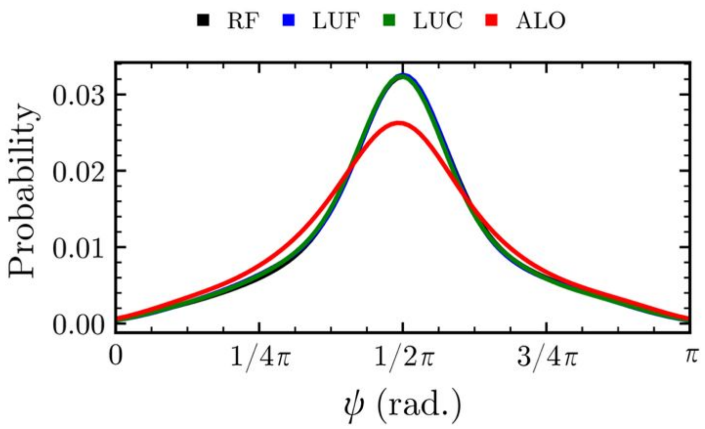

Figure S9. Probability distributions of the parameter ψ for HU^- dimers in the vicinity of riboflavin (black), lumiflavin (blue), lumichrome (green), and alloxazine (orange).

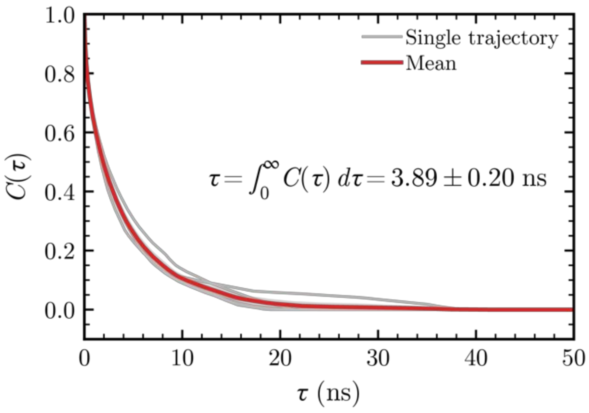

Figure S10. Bond-lifetime autocorrelation analysis for HU- contacts with riboflavin. The time correlation function C(tau) was computed for modifier-HU- contacts using multiple time origins over five independent trajectories. The characteristic contact lifetime extracted from the correlation analysis is 3.89 ± 0.20 ns.

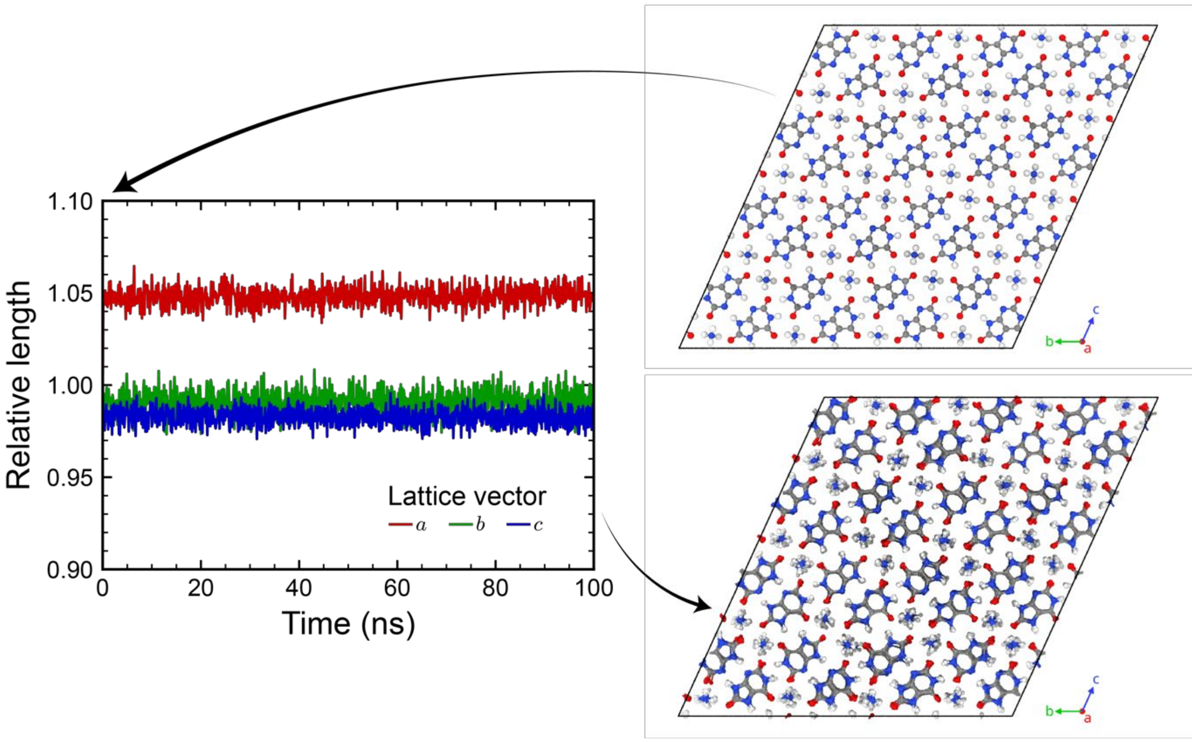

Figure S11. Validation of the NH4HU crystal structure described by the force field. Left: time evolution of the lattice parameters a, b, and c relative to the experimental values during an unconstrained 100 ns NPT simulation at 300 K and 1 bar. Right: representative crystal configurations at the beginning and end of the simulation, showing that the molecular packing is maintained. The small deviations of the lattice parameters from experiment support the suitability of the force field for describing crystal-derived fingerprints.

Table S1. Parameters for crystal fingerprint and crystal-conforming conformers.

$r_{\text{fingerprint}}$	$r_{\text{conformers}}$	$\theta_{\text{fingerprint}}$	$\theta_{\text{conformers}}$	$\psi_{\text{fingerprint}}$	$\psi_{\text{conformers}}$
4.3	4.2 ± 0.25	π	$\pi \pm 1/16\pi$	π	$\pi \pm 1/8\pi$
7.5	7.7 ± 0.25	π	$\pi \pm 1/16\pi$	π	$\pi \pm 1/8\pi$
8.0	8.6 ± 0.25	π	$\pi \pm 1/16\pi$	π	$\pi \pm 1/8\pi$

Movies

Movie 1. Molecular dynamics trajectory corresponding to Figure 3C, showing RF directly capturing two HU^- molecules to form a coplanar HU^- dimer.

Movie 2. Molecular dynamics trajectory corresponding to Figure 3D, illustrating RF-promoted conversion of a stacked HU^- dimer into a coplanar dimer.

Movie 3. Trajectories of two HU^- molecules in the vicinity of RF, showing the dynamic interconversion of hydrogen-bonding patterns between HU^- molecules.

References

1. W. Tang, *et al.*, Tautomerism unveils a self-inhibition mechanism of crystallization. *Nat Commun* **14**, 561 (2023).

2. M. Warzecha, *et al.*, Olanzapine crystal symmetry originates in preformed centrosymmetric solute dimers. *Nat. Chem.* **12**, 914–920 (2020).

3. M. J. Abraham, *et al.*, GROMACS: High performance molecular simulations through multi-level parallelism from laptops to supercomputers. *SoftwareX* **1–2**, 19–25 (2015).

4. X. He, V. H. Man, W. Yang, T.-S. Lee, J. Wang, A fast and high-quality charge model for the next generation general AMBER force field. *J Chem Phys* **153**, 114502 (2020).

5. C. W. Hopkins, S. Le Grand, R. C. Walker, A. E. Roitberg, Long-Time-Step Molecular Dynamics through Hydrogen Mass Repartitioning. *J. Chem. Theory Comput.* **11**, 1864–1874 (2015).

6. C. Balusek, *et al.*, Accelerating Membrane Simulations with Hydrogen Mass Repartitioning. *J. Chem. Theory Comput.* **15**, 4673–4686 (2019).

7. W. Humphrey, A. Dalke, K. Schulten, VMD: Visual molecular dynamics. *Journal of Molecular Graphics* **14**, 33–38 (1996).

8. E. C. Meng, *et al.*, UCSF ChimeraX: Tools for structure building and analysis. *Protein Science* **32**, e4792 (2023).

9. B. Duan, *et al.*, Comparative studies on structure, sensitivity and mechanical properties of CL-20/DNDAP cocrystal and composite by molecular dynamics simulation. *RSC Advances* **8**, 34690–34698 (2018).

10. X. Li, L. Song, Y. Zhao, X.-H. Ju, Crystal morphology prediction of CL-20 and 1,4-DNI co-crystal at different temperatures. *J Mol Model* **29**, 135 (2023).

11. M. H. Shariare, F. J. J. Leusen, M. de Matas, P. York, J. Anwar, Prediction of the Mechanical Behaviour of Crystalline Solids. *Pharm Res* **29**, 319–331 (2012).

12. M. J. Frisch, *et al.*, Gaussian 09 revision a.2. (2009).

13. T. Lu, A comprehensive electron wavefunction analysis toolbox for chemists, Multiwfn. *J. Chem. Phys.* **161**, 082503 (2024).

14. W. Tang, C. Smith, C. B. Parry, J. Meegan, J. D. Rimer, Molecular Imposters Functioning as Versatile Growth Modifiers of Urate Crystallization. *Crystal Growth & Design* **23**, 6107–6118 (2023).
