# Supplementary figures and images for: Mechanistic Insights into Molecular Modifiers That Promote Urate Crystallization through Solute Assembly Regulation
Source: JACS Au. 2026 Apr 17;6(5):2819–26. doi: 10.1021/jacsau.6c00140 (PMC13213513; doi:10.1021/jacsau.6c00140)

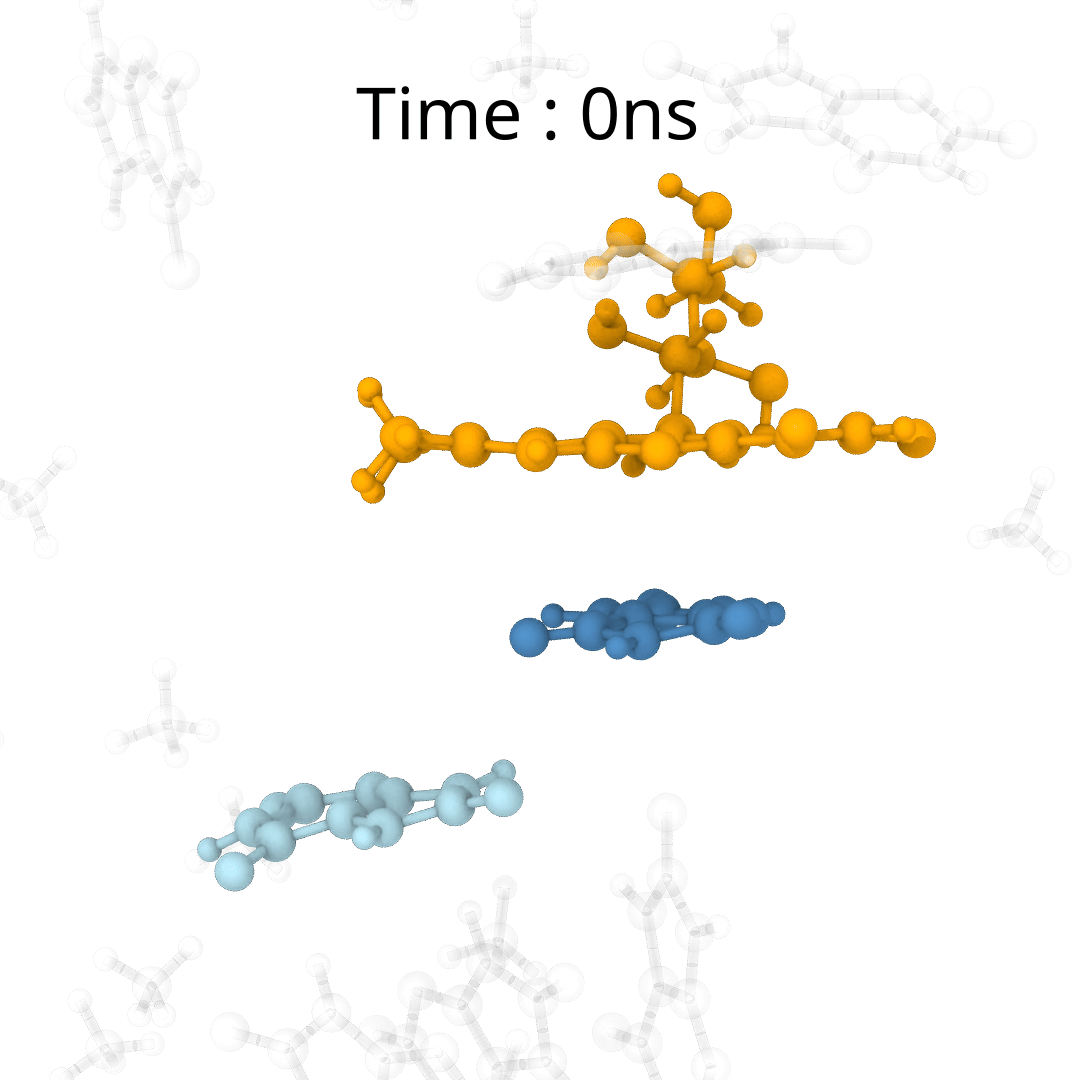

Supplement: Supplementary file 2 [file au6c00140_si_002.gif]

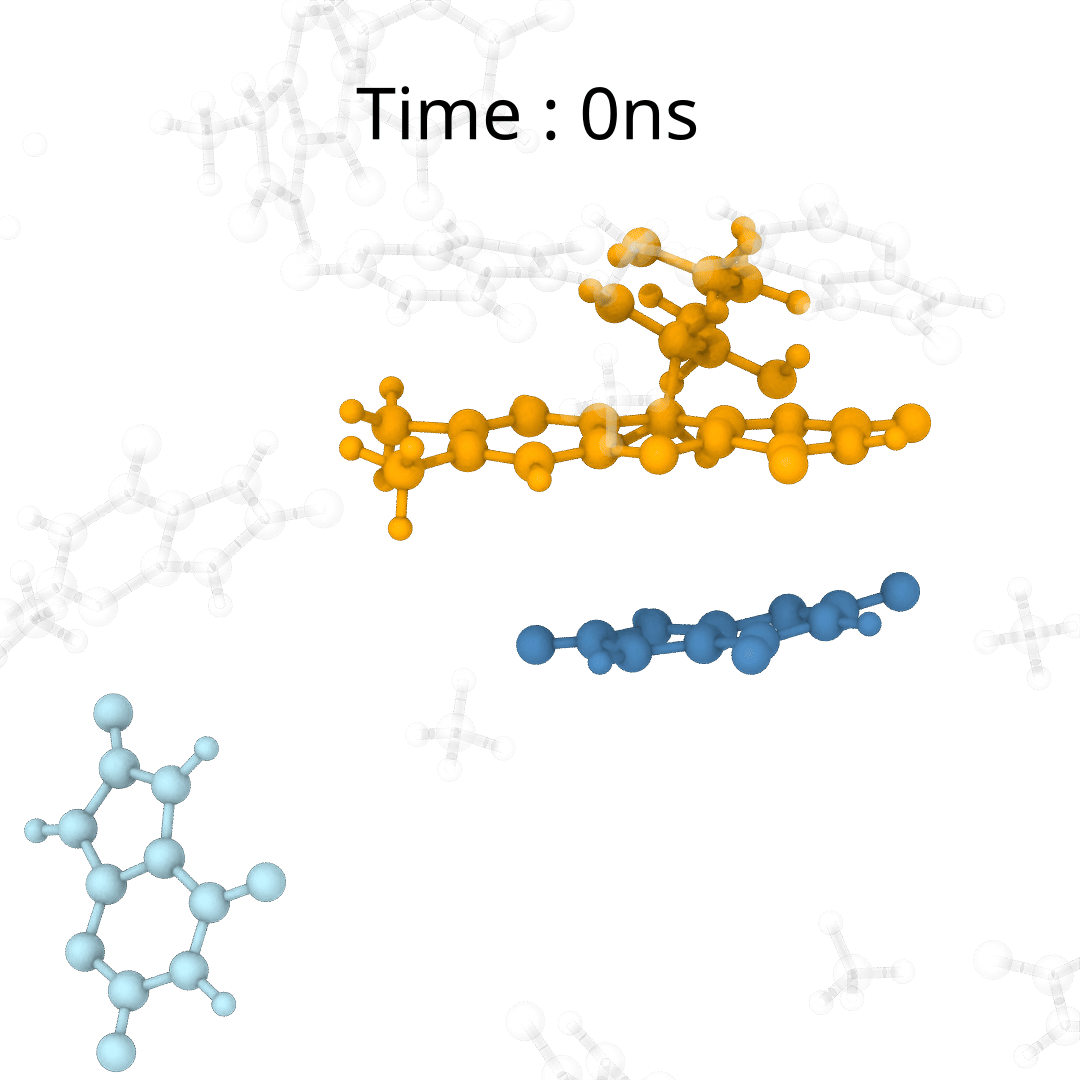

Supplement: Supplementary file 3 [file au6c00140_si_003.gif]

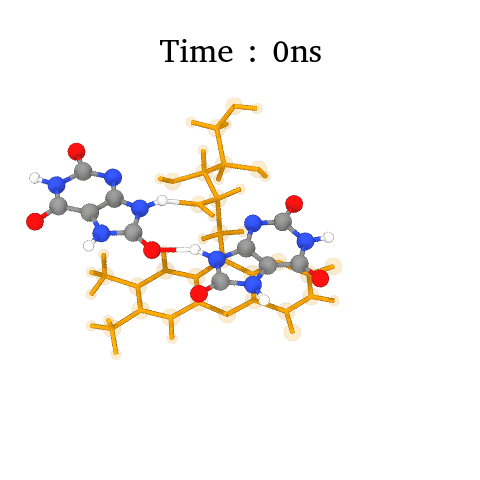

Supplement: Supplementary file 4 [file au6c00140_si_004.gif]
